# Strategies to Encapsulate the *Staphylococcus aureus* Bacteriophage phiIPLA-RODI

**DOI:** 10.3390/v10090495

**Published:** 2018-09-13

**Authors:** Eva González-Menéndez, Lucía Fernández, Diana Gutiérrez, Daniel Pando, Beatriz Martínez, Ana Rodríguez, Pilar García

**Affiliations:** 1 Instituto de Productos Lácteos de Asturias (IPLA-CSIC), Paseo Río Linares s/n, 33300 Villaviciosa, Spain; eva.gm@ipla.csic.es (E.G.-M.); lucia.fernandez@ipla.csic.es (L.F.); dianagufer@ipla.csic.es (D.G.); bmf1@ipla.csic.es (B.M.); anarguez@ipla.csic.es (A.R.); 2 Nanovex Biotechnologies S.L., Parque Tecnológico de Asturias, CEEI, 33428 Llanera, Spain; pando@nanovexbiotech.com

**Keywords:** bacteriophages, encapsulation, niosomes, transfersomes, liposomes, *Staphylococcus aureus*

## Abstract

The antimicrobial properties of bacteriophages make them suitable food biopreservatives. However, such applications require the development of strategies that ensure stability of the phage particles during food processing. In this study, we assess the protective effect of encapsulation of the *Staphylococcus aureus* bacteriophage phiIPLA-RODI in three kinds of nanovesicles (niosomes, liposomes, and transfersomes). All these systems allowed the successful encapsulation of phage phiIPLA-RODI with an efficiency ranged between 62% and 98%, regardless of the concentration of components (like phospholipids and surfactants) used for vesicle formation. Only niosomes containing 30 mg/mL of surfactants exhibited a slightly lower percentage of encapsulation. Regarding particle size distribution, the values determined for niosomes, liposomes, and transfersomes were 0.82 ± 0.09 µm, 1.66 ± 0.21 µm, and 0.55 ± 0.06 µm, respectively. Importantly, bacteriophage infectivity was maintained during storage for 6 months at 4 °C for all three types of nanovesicles, with the exception of liposomes containing a low concentration of components. In addition, we observed that niosomes partially protected the phage particles from low pH. Thus, while free phiIPLA-RODI was not detectable after 60 min of incubation at pH 4.5, titer of phage encapsulated in niosomes decreased only 2 log units. Overall, our results show that encapsulation represents an appropriate procedure to improve stability and, consequently, antimicrobial efficacy of phages for application in the food processing industry.

## 1. Introduction

Bacteriophages, viruses that infect and kill bacteria, are ubiquitous in the environment. Indeed, they are considered to be the most abundant organisms in the biosphere, playing an important role in biogeochemical cycles and in the development of microbial communities [1]. Over the last few years, there has been a notable interest in exploiting the antimicrobial properties of phages for the control of bacterial pathogens (phage therapy), as a strategy to curtail the relentless increase in antibiotic-resistant bacteria [2,3,4]. Moreover, phages are harmless to humans, animals, and plants, making them a safe alternative to conventional antimicrobials [5].

Another potential application of bacteriophages is as biocontrol agents along the food chain [6]. The main advantages of bacteriophages in a food context are related to their specificity and safety, and several studies have already confirmed their effectiveness. For example, bacteriophages infecting the most important foodborne pathogens have been successfully used to reduce the microbial load in livestock, thereby decreasing the risk of transmission of zoonotic bacteria. Also, phages can be directly applied to foods as preservatives to prevent the development of undesirable bacteria [7,8,9]. Additionally, bacterial viruses have been proposed as promising disinfectants to reduce the risk of food contamination caused by biofilms in food processing industries [10]. Nonetheless, widespread use of bacteriophages in agriculture, farming or food industrial settings will not be possible until they receive full approval by the regulatory authorities. In that sense, it is worth mentioning the recent approval of several phage-based products by the U. S. Food and Drug Administration to be used as food preservatives. Thus, two companies, Intralytix, Inc. (http://www.intralytix.com) and Micreos (http://www.micreos.com) currently market an array of phage-based products against some of the most important foodborne pathogenic bacteria. Some examples include *Listeria monocytogenes* (ListShield™ and PhageGuard Listex), *E. coli* O157:H7 (EcoShield™), *Shigella* (ShigaShield™) and *Salmonella* (SalmoFresh™ and PhageGuard S).

Depending on the specific use, phages can be delivered by oral administration to animals or by direct spraying on food or industrial surfaces [7,11,12]. In many cases, bacteriophages will encounter harsh physicochemical conditions following the application of phage-based products. For instance, the presence of bile salts and low pH in the gastrointestinal tract of farm animals or the UV light and low pH on the surface of some fruits may lead to inactivation of the viral particles. This would unavoidably result in a loss of infectivity against their target bacterium and, ultimately, failure of the disinfection procedure.

A feasible possibility to overcome this problem is the encapsulation of phages to protect them from environmental challenges so that they can reach their target microbes. (Micro- or nano-) encapsulation is a technology that allows packaging solid, liquid, or gaseous materials in miniature capsules or vesicles that can release their contents at controlled rates, sometimes triggered by specific environmental cues (pH, temperature, etc.) [13]. Indeed, this technique is often used in the food industry for the delivery of bioactive compounds or probiotics [14]. Thus, nanoencapsulation of different compounds like vitamins, minerals, and proteins confers several advantages since the molecules inside the nanovesicles are protected from hazardous environmental conditions and display increased stability as well as greater intestinal and epidermal absorption [15,16,17,18]. Indeed, there are several examples in literature where microencapsulation increased the stability of probiotic bacteria in dairy products [19], or the viability of these bacteria during their passage through the gastrointestinal tract [20,21]. Similarly, nanoencapsulation of an antimicrobial peptide (nisin) or lysozyme allowed overcoming stability issues and prevented their interaction with food components when used as additives for dairy products to control the growth of pathogenic bacteria [22,23]. Thus far, there are some studies regarding the encapsulation of bacteriophages in emulsions as well as in micro and nanovesicles prepared using different techniques based on chemical (polymerization) or physical (drying or extrusion) processes. However, there is only limited information about the encapsulation of bacteriophages in liposomes and none concerning the use of other types of nanovesicles (reviewed by [24,25]).

*Staphylococcus aureus* is an important pathogenic bacterium responsible for serious infections and food-borne diseases in humans [26,27]. The European Food Safety Authority (EFSA) reported a total of 434 food-borne outbreaks caused by staphylococcal toxins in 2015 [28]; meanwhile, 360 outbreak-associated illnesses and 27 hospitalizations were caused by *S. aureus* toxins in the US in 2016 [29]. Furthermore, the increase in methicillin-resistant (MRSA) and vancomycin-resistant strains (VRSA) has fostered research on the development of new weapons to fight against this pathogen [30]. Among these weapons, bacteriophages and phage-derived proteins are an interesting alternative to treat *S. aureus* infections [31,32] and prevent food contamination [33,34].

Our previous work had already demonstrated the ability of bacteriophages to reduce *S. aureus* contamination in dairy products including milk, curd, and cheese [35,36,37]. Moreover, there is evidence that they can also be used as a helper when combined with hydrostatic high pressure (HHP) to improve the safety of dairy products [38]. More recently, we have isolated and characterized the polyvalent *Myoviridae* phage phiIPLA-RODI, which is able to infect a broad range of staphylococcal species including *S. aureus* strains from food industry origin [39]. Its high infectivity against both planktonic cultures and biofilms makes it a good candidate for the biocontrol of *S. aureus* in food-related settings. However, the poor stability of phiIPLA-RODI under certain environmental conditions, might compromise its use during processing of fermented products.

The aim of this study was to evaluate the stability of phage phiIPLA-RODI under different encapsulation conditions compatible with the delivery of phage to foods. We also assessed the viability of the encapsulated phage during storage as well as its resistance under extreme conditions.

## 2. Materials and Methods

### 2.1. Bacterial Strains

*S. aureus* IPLA1 was used as the host strain of phage phiIPLA-RODI [39]. Isolated colonies of this strain were obtained by streaking out a frozen stock onto Baird–Parker (BP) agar plates. Subsequent bacterial cultures were routinely grown in tryptic soy broth (TSB; Scharlau, Barcelona, Spain) with shaking or on plates containing TSB supplemented with 2% (*w*/*v*) bacteriological agar (TSA). The growth temperature for all experiments was 37 °C.

### 2.2. Phage Propagation and Enumeration

Bacteriophage phiIPLA-RODI was routinely propagated as previously described [39]. Briefly, early exponential cultures of *S. aureus* IPLA1 (OD_600_ = 0.1) were infected with the phage at a multiplicity of infection (MOI) of 1. The infected cultures were then incubated for 3 h at 37 °C with vigorous shaking. Phage lysates were obtained by centrifugation of these cultures and subsequent filtration (0.45 µm cellulose acetate Sartolab^®^ RF Vacuum Filters). Afterwards, the phage lysate was partially purified by adding NaCl (0.5 M, final concentration) and PEG 8000 (10%, final concentration). These samples were incubated at 4 °C for 24 h, and then centrifuged at 10,000 rpm at 4 °C for 30 min. The pellet containing the phage particles was suspended in SM buffer (20 mM Tris HCl, 10 mM MgSO_4_, 10 mM Ca(NO_3_)_2_ and 0.1 M NaCl, pH 7.5).

Phage titer was calculated by the double-layer plaque assay. Briefly, 0.1 mL from a 1:10 dilution of a *S. aureus* IPLA1 stationary culture (10^8^ CFU/mL) were mixed with several dilutions of individual phage suspensions in 3 mL of molten TSB supplemented with 0.7% agar and the mixture was poured onto TSA plates and incubated at 37 °C for 18 h. The phage titer was expressed as PFU/mL. All assays were performed using biological triplicates and values were presented as the mean ± standard deviation.

### 2.3. Encapsulation Processes

Liposomes are bilayer vesicles prepared with phospholipids and containing cholesterol as a stabilizing agent. Their size ranges depending on the type of vesicles formed are the following: multilamellar vesicles (size > 200 nm), large unilamellar vesicles (100–400 nm), small unilamellar vesicles (<100 nm).

Niosomes and transfersomes are similar to liposomes in terms of structure and physical properties. They can be unilamellar or multilamellar vesicles prepared by adding surfactants and other amphiphilic molecules, alternative to phospholipids, such as non-ionic surfactants [40].

Liposomes and niosomes were prepared using Pronanosome Lipo-N^TM^ and Pronanosome Nio-N^TM^ (Nanovex Biotechnologies, Llanera, Spain), respectively. Briefly, different amounts of Pronanosomes were employed to reach different final concentrations (30, 50 and 70 mg/mL). The products were hydrated with a solution of 0.05 M HEPES buffer pH 7.5 (Alfa Aesar, Karlsruhe, Germany) and then the bacteriophage suspension (3.2 × 10^8^ PFU/mL) was added at a final concentration of 4% (1.3 × 10^7^ PFU/mL). Then, the samples were mixed in two steps: a first step of manual shaking for 3 min followed by a homogenization step using a homogenizer (Heidolph, Schwabach, Germany) at different speeds and times (Table 1).

Transfersomes were prepared by the thin film hydration (TFH) or dry film method [41] with minor modifications. Different amounts (30, 50 and 70 mg/mL) of Phospholipon 90G (P90; Lipoid, Ludwigshafen, Germany) and Span 60 (Alfa Aesar, Karlsruhe, Germany) (1:1) were dissolved in chloroform. Chloroform was subsequently removed by incubation at 40 °C, under reduced pressure in a rotary evaporator (Heidolph, Schwabach, Germany) until the formation of a dry film, which was subsequently hydrated by a solution of 0.05 M HEPES buffer pH 7.5. Then, bacteriophages were added at a final concentration of 4% (1.3 × 10^7^ PFU/mL). The nanovesicles were subsequently generated after being submitted to 3 min of manual shaking and homogenized for 5 min at 8000 rpm (Table 1).

### 2.4. Characterization of Nanovesicles

Mean (Z-Average) particle size in the different nanovesicles (niosomes, liposomes and transfersomes) was determined by using a Zetasizer Nano ZS (Malvern Instruments Ltd., Worcestershire, UK), via Dynamic Light Scattering (DSL).

The ζ-potential is strongly linked to vesicle stability, with high absolute values of ζ-potential indicating electrostatic repulsion between vesicles. To determine the ζ-potential the M3-PALS (Phase Analysis Light Scattering) technique was used [15]. Three independent samples were taken from each formulation at room temperature and values were expressed as mean ± standard deviation.

### 2.5. Viability and Encapsulation Efficiency

The number of bacteriophages that did not survive the formulation process for niosomes, liposomes or transfersomes, was expressed as log reduction in viability:

(1) $$\mathrm{Viability}\;={\;\log}_{10}\left(\frac{\mathrm{Total}\;\mathrm{phage}\;\mathrm{titer}\;\mathrm{after}\;\mathrm{treatment}\;\left(\frac{\mathrm{PFU}}{\mathrm{mL}}\right)}{\mathrm{Initial}\;\mathrm{titer}\;\left(\frac{\mathrm{PFU}}{\mathrm{mL}}\right)}\right)$$

Phage viability after the formation of niosomes, liposomes and transfersomes was obtained by determining the total phage titer (encapsulated + non encapsulated). To determine the titer of non-encapsulated phages or free phages (F), aliquots containing 1.5 mL of niosomes, liposomes and transfersomes were centrifuged at 13,200 rpm and 4 °C for 60 min and the supernatant was titrated as described above. To determine the titer of encapsulated phages, the pellet containing the nanovesicles was washed twice with PBS buffer (137 mM NaCl, 2.7 mM KCl, 10 mM 138 Na_2_HPO_4_ and 2 mM KH_2_PO_4_; pH 7.4) following supernatant removal, and centrifuged again under the same conditions. Again, the supernatant was removed and the pellet was kept for further processing. Then, pellets were treated with 30 µL of chloroform and vortexed for 5 s to disrupt the vesicles. Finally, SM buffer was added up to a final volume of 1.5 mL. This mixture was centrifuged for 15 min at 10,000 rpm and 4 °C. Serial dilutions of the supernatant were then plated by using the double layer technique for phage titration.

The efficiency of encapsulation (EE) was calculated as the percentage of phages encapsulated inside nanovesicles compared to the total phage titer:

(2) $$\mathrm{EE}\;=\left(\frac{\mathrm{Encapsulated}\;\mathrm{phage}\;\left(E\right)\;\left(\mathrm{PFU}/\mathrm{mL}\right)}{\mathrm{Total}\;\mathrm{phage}\;\left(\mathrm{PFU}/\mathrm{mL}\right)}\right)\;\times 100$$

where, total phage = encapsulated phage (E) + non encapsulated or free phage (F).

### 2.6. Stability of Encapsulated Phages During Storage

The stability of phages entrapped into nanovesicles (niosomes, liposomes, and transfersomes) was determined by storage of samples at 4 °C for 6 months. Every two months the viability of free and encapsulated phages was determined. A phiIPLA-RODI suspension (1.49 × 10^8^ PFU/mL) in SM was used as control. Three independent samples were taken from each formulation and from the control phage suspension. The titer was presented as the mean of these values ± standard deviation.

### 2.7. Stability of Encapsulated Phages Under Extreme Conditions

The stability of phages encapsulated in niosomes, liposomes, and transfersomes at a pH of 4.5 was determined by preparing a 1:2 dilution of the nanovesicles in Britton–Robinson pH universal buffer (40 mM H_3_PO_4_, 40 mM CH_3_COOH, 40 mM H_3_BO_3_, adjusted to pH 4.5) and then incubating the samples at room temperature for 60 min. Similarly, nanovesicles were incubated in the following conditions: (1) dilution (1/10) in NaCl 4.5 M for 60 min at room temperature, and (2) at 60 °C for 90 min. For control purposes, a phage suspension in SM buffer was subjected to the same treatments. After treatment, samples were centrifuged and the supernatant was titrated to determine the number of non-encapsulated phages. Viability of encapsulated phages was quantified after disruption of the nanovesicles as indicated above. The initial titer of samples before treatment was taken as a control. All assays were performed by triplicate.

### 2.8. Statistical Analysis

Statistical analyses for encapsulation efficiency and phage stability were performed using the statistical package IBM SPSS Statistics for Windows Version 23 (IBM Corp., Armonk, NY, USA). The differences in data related to phage encapsulation efficiency and its stability were subjected to one-way analysis of variance (ANOVA). The Student–Newman–Keuls (SNK) test was used for comparison of means of the stability of phiIPLA-RODI in the different nanovesicles at a level of significance *p* < 0.05. Three biological replicates were used in all the assays.

## 3. Results

### 3.1. Bacteriophage phiIPLA-RODI Can Be Successfully Entrapped in Different Nanovesicles

With the aim of seeking an optimal formulation for the application of phage phiIPLA-RODI, we examined the efficacy of different encapsulation techniques. In a first step, we determined the stability of the phage during different encapsulation processes and assessed the properties of the resulting nanovesicles. To do that, a phage suspension (3.2 × 10^8^ PFU/mL) was encapsulated into niosomes, liposomes, and transfersomes. Then, the size of the prepared vesicles was measured using Dynamic Light Scattering. The nanovesicles formed had an average size in the range of 0.5 μm to 2 μm (0.82 ± 0.09 µm for niosomes, 1.66 ± 0.21 µm for liposomes and 0.55 ± 0.06 µm for transfersomes). Also, the net charge of the nanovesicles surface was estimated by determination of the ζ-potential, with liposomes showing the lowest absolute values (Table 1). Additionally, the viability of phages after the encapsulation process was determined. The results obtained in phage titration assays indicated that phiIPLA-RODI phage particles could withstand all the encapsulation methods tested, as all nanovesicles contained infective phages. However, the titer of phages released after the disruption of nanovesicles was variable, with a reduction ranging from 0.5 to 1.4 log units compared to the initial phage titer (Table 1).

### 3.2. The Efficiency of phiIPLA-RODI Encapsulation Is Not Greatly Influenced by Component Concentration

Once established that phage phiIPLA-RODI could be successfully encapsulated in nanovesicles, we examined whether differences in the composition of the vesicles may affect the encapsulation efficiency. In order to do that, the three types of nanovesicles (niosomes, liposomes and transfersomes) were prepared by using different concentrations (30, 50 and 70 mg/mL) of their respective components. The results of this experiment indicated that the percentage of encapsulation was largely independent of the concentration of components or the nanovesicle type (*p* > 0.05) (Table 2). The only exception to this was that, for niosomes, the highest encapsulation efficiencies (99% and 94%) were obtained by using 30 and 50 mg/mL of Pronanosome Nio-N™, while only a 62% was observed when niosomes were made with 70 mg/mL. Although not statistically significant, it is worth noting that liposomes formed using 70 mg/mL of Pronanosome Lipo-N™ and transfersomes containing 30 mg/mL of components (Phospholipon 90G and Span 60) showed variable results between different replicates (Table 2). This suggests that encapsulation of phiIPLA-RODI using those concentrations would not consistently result in high encapsulation efficiencies.

### 3.3. Stability of Encapsulated Phage Particles during Storage at Low Temperature

One of the main challenges associated to the development of phage-based products is to ensure their stability under storage conditions. With this in mind, we assessed whether encapsulated and non-encapsulated phiIPLA-RODI particles remained viable during storage at 4 °C throughout a 6-month period. As a control, we tested the stability of a phage suspension in SM buffer (1.49 × 10^8^ PFU/mL).

**Figure 1:**
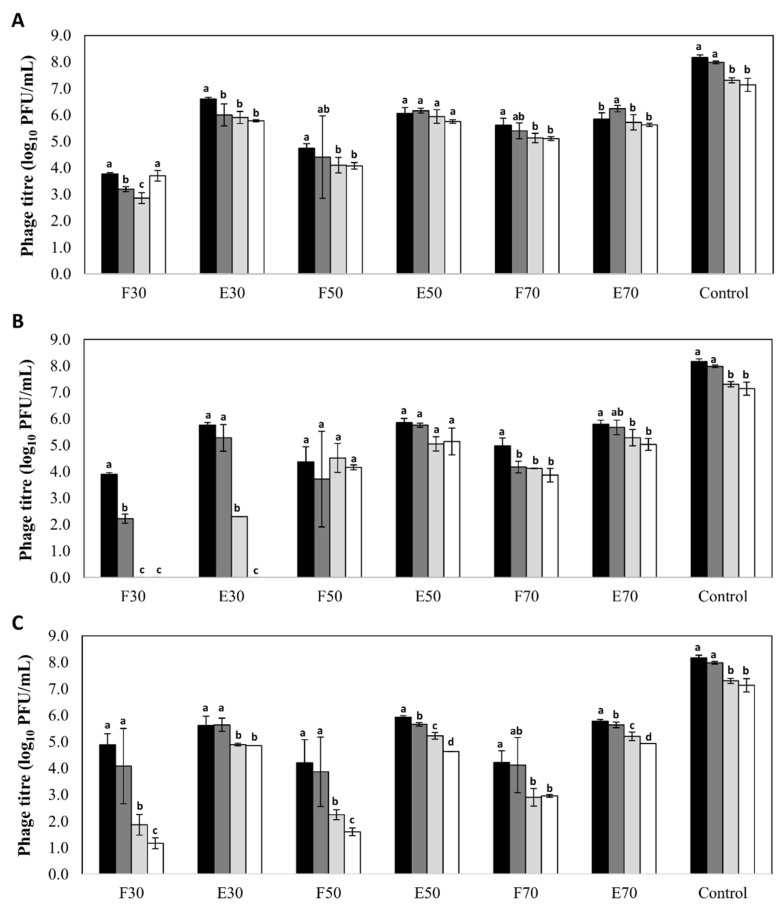
Stability (log_10_ PFU/mL) of bacteriophage phiIPLA-RODI encapsulated in different types of nanovesicles and in SM buffer (control), after storage at 4 °C: (**A**) niosomes, (**B**) liposomes and (**C**) transfersomes. Phage titer was determined after encapsulation (black bars) and also after storage for 2 months (dark grey bars), 4 months (light grey bars) and 6 months (white bars). (F): non encapsulated or free phage; (E): encapsulated phage. Numbers (30, 50 or 70) indicates the concentration of components expressed in mg/mL. Bars represent mean ± standard deviation of three biological replicates. Different letters indicate differences in stability (*p* < 0.05; ANOVA and SNK post-hoc comparison).

A high stability was observed for phages encapsulated in all three types of nanovesicles regardless of the nature and concentration of their components, with decreases in phage titer below 2 log units (Figure 1). The only exception was observed for phages encapsulated in liposomes formed with 30 mg/mL of Pronanosome Lipo-N^TM^. In this case, there was a 3-log reduction in encapsulated phage titer after four months, and a similar decrease in non-encapsulated phage titer after only two months. In general, the stability of free phages was lower than that of encapsulated phages in all the formulations tested. Indeed, in the case of transfersomes, the differences in phage titer after six months between the encapsulated and non-encapsulated fractions ranged between 2 and 4 log units depending on the component concentration (Figure 1). In contrast, the differences observed for niosomes and liposomes were always below 1 log unit (Figure 1). Surprisingly, the titer of control phage suspension was reduced only by 1 log unit (7.14 ± 0.25 log_10_ PFU/mL) after 6 months of storage, which means that, only for phages inside niosomes (50 and 70 mg/mL) and liposomes (50 mg/mL), the stability of phage was improved (*p* < 0.05).

### 3.4. Niosomes Protect Phages from Low pH and High Temperature

A major advantage of nanovesicles for the application of bacteriophages in the food industry would be the protection of the viral particles from adverse physicochemical conditions commonly found in food-processing settings. Here, we examined if encapsulation of the phiIPLA-RODI in niosomes, liposomes or transfersomes had a protective effect against low pH. The results obtained indicated that niosomes effectively protect phages at a pH of 4.5. Thus, while non-encapsulated phages and control phage suspension were completely inactivated under these conditions, phages entrapped inside niosomes retained part of their infectivity, exhibiting only a reduction of ~2 log units (Table 3).

Overall, liposomes turned out to be less stable at low pH values, as only those vesicles containing 50 and 70 mg/mL of Pronanosome Lipo-N™ were able to protect the encapsulated phages, showing a reduction of nearly 2–3 log units with respect to the initial phage titer. No protective effect from low pH was achieved in the case of transfersomes, as no active phage particles could be detected after the treatment (Table 3).

Regarding temperature stability, phage encapsulation in liposomes and transfersomes did not offer protection in treatments at 60 °C for 90 min. Indeed, a total loss of viability was observed for both encapsulated and non-encapsulated phages. Similarly, an equivalent reduction in phage titer (about 4 log units) was recorded for the control phage suspension. In contrast, niosomes exerted better protection of the encapsulated phages since lower reduction in phage titer (2–3 log units) was detected (Table 3).

Although high NaCl concentrations had not any appreciable effect on phage stability, we tested the impact on nanovesicles of 4.5 M NaCl for 60 min, since this salt concentration could cause vesicles destabilization. Indeed, we observed a slight reduction (~1 log unit) in encapsulated phage titer and an increase in non-encapsulated phages, regardless of the type of nanovesicle and the surfactant concentration used (Table 3). These results suggest a slight destabilization of the nanovesicles in the presence of a high salt concentration that results in the partial release of encapsulated phages.

## 4. Discussion

The increasing number of studies confirming the success of phages as antimicrobials along with the current trend in the consumption of healthy, chemical-free foods has boosted the interest in bacteriophages as natural biopreservatives [42] and disinfectants [10]. However, for phage-based products to be successful, it is necessary to design proper formulations that meet certain stability requirements that standard phage suspensions do not currently have. Microencapsulation techniques including emulsification, extrusion, spray-drying, electrospun nanofibers and whey protein films have been proposed as feasible alternatives to solve this problem [24]. In addition, the use of food-compatible nanomaterials together with an approved preparation procedure, including food-grade solvents and detergents, is also an essential requirement [14]. In the present study, we attempted to find the most effective techniques for the encapsulation of phage particles to be used in food industry applications.

In a first step, we confirmed that phage phiIPLA-RODI remained stable after different encapsulation processes including niosomes, liposomes and transfersomes.

Phages encapsulated into niosomes (50 and 70 mg/mL of Pronanosome Nio-N™) and liposomes (50 mg/mL of Pronanosome Lipo-N™) retained stability for longer periods of time than those kept in SM suspension. All studies published so far report the use of liposomes as vehicles to encapsulate phages [8,43], but there are no reports about the use of niosomes and transfersomes. To determine the optimal conditions that result in the highest encapsulation efficiency for phage phiIPLA-RODI, we prepared nanovesicles using different concentrations of surfactants and phospholipids. A previous study reported that total lipid concentration was an important factor for the loading efficiency of liposomes [44]. However, we did not usually observe this trend when encapsulating phiIPLA-RODI in different types of nanovesicles. Indeed, a reduction in component concentration only improved encapsulation of phiIPLA-RODI into niosomes, but did not significantly modify the encapsulation efficiency in liposomes or transfersomes. Additionally, we could not establish any correlation between encapsulation efficiency and the average particle size associated to each vesicle type. For instance, the average size of transfersomes (~0.5 µm) was three times smaller than that of liposomes (~1.6 µm), but similar phage encapsulation efficiencies were obtained with both types of nanovesicles. In contrast, Leung et al. [45], found that encapsulation efficiency increased with vesicle size in liposomes obtained by using microfluidics, although this trend was only observed for small vesicles (up to 0.5 µm).

The use of encapsulated phages for therapeutic [25] or food applications [46] requires optimization of the formulation process. For example, it is paramount to control the release of phages from nanovesicles and maximize the stability of the encapsulated phage particles. Release of phages can be controlled by external parameters such as temperature or pH, provided they do not negatively affect the phage, or by spontaneous rupture in contact with components of food matrix [25]. Overall, we found that the stability during storage of the encapsulated phages was always higher than that of free or non-encapsulated phages in the same suspension, but the stability was only higher than that of the control phage suspension in SM buffer in some cases. A high stability of encapsulated phages is in accordance (consonance) with previous findings where nanoencapsulation in liposomes increased phage stability during storage conditions as well as during their delivery to animals [8,43]. Moreover, the use of phages inside liposomes facilitates their entry into macrophages and also protects phages from neutralizing antibodies [47]. More specifically, we found that encapsulation of phage phiIPLA-RODI into niosomes and liposomes led to a slightly higher stability during storage at 4 °C than encapsulation into transfersomes. Thus, viability of phages encapsulated in transfersomes (70 mg/mL of Phospholipon 90G and Span 60) after six months of storage was lower than that obtained for niosomes. It does not appear that the low stability of phages in transfersomes is related with the ζ-potential of these nanovesicles, as it was quite similar to that of niosomes. It is also worth noting the deleterious effect for phage phiIPLA-RODI of storage in liposomes containing 30 mg/mL of Pronanosome Lipo-N™. In fact, even non-encapsulated phages were inactivated in these suspensions. It does not seem, however, that this result is due to a low stability of phiIPLA-RODI at low temperatures since this phage is highly stable under refrigeration conditions. We can speculate that components from liposomes might bind to the phage proteins necessary for the recognition of and/or binding to the cell receptor, thereby inhibiting their interaction with host bacteria. An increase in the concentration of components might help to stabilize liposomes, as can be deduced from the higher protection of phages by liposomes containing 50 and 70 mg/mL of Pronanosome Lipo-N^TM^. Therefore, the low stability of liposomes containing 30 mg/mL would result in the release of these components to the suspension. These components may interact with phage proteins and potentially inactivate the phage particles.

Despite the well-established antimicrobial activity of bacteriophages in some food applications, their use might be limited under certain processing conditions that inactivate the phage particles. For instance, the fermentation of milk by lactic acid bacteria results in a pH ≤ 4.5, which leads to the inactivation of many bacteriophages including phiIPLA-RODI [39]. For this reason, we investigated here if encapsulation was an effective method for the protection of phage phiIPLA-RODI from low pH values. Our results confirmed the protective effect of niosomes under acidic conditions. However, we observed that liposomes only offered partial protection to the phage particles, with a decrease in phage viability of up to 3 log units. Regarding transfersomes, our results indicated that they had a negligible contribution to phage stability maintenance. Moreover, our results show that transfersomes themselves were not stable at low pH values, which may be related to their specific content in Phospholipon 90G and Span 60. In fact, it is a common practice to modulate liposome stability at different pH values by changing their composition as a means to control the release of the vesicle content [48]. This has great relevance for the delivery of drugs, enzymes, and other therapeutics into vesicles that will only release their content at the pH of the target organ [48]. In an agro-food context, protection of phages against extremely acidic conditions was an approach used by Colom [8] to deliver *Salmonella* phages to the chicken stomach and to increase their residence time in the intestinal tract. These authors found that phages encapsulated into liposomes were stable after exposure to a pH of 2.8 and, although the phage titer was reduced by 3–5 log units, protection against *Salmonella* by the encapsulated phages persisted for at least 1 week.

In relation to the higher stability to temperature of niosomes, their composition with non-ionic surfactants might explain this result because they are more stable than phospholipids [49]. Indeed, phage phiIPLA-RODI inside niosomes turned out to be less sensitive to temperature than in a SM buffer suspension, as a reduction of 2–3 log-units was observed compared to 4 log units reduction in the control suspension.

Furthermore, we explore the stability of nanovesicles under external conditions that, without having an effect on phage viability, might destabilize their structure and, therefore, expose phages to further undesirable conditions. For example, high salt concentrations are frequently used in the processing of some foods such as cheese, but do not seem to affect the viability of phiIPLA-RODI. Indeed, all nanovesicles turned out to be quite stable in the presence of NaCl. However, in some cases, we observed a slight increase in non-encapsulated phages and a reduction in encapsulated phages, which suggests that some nanovesicles opened spontaneously, releasing the encapsulated phage particles. The spontaneous release of compounds when nanovesicles are applied to food might be a feasible approach for phage delivery. It has been previously used to incorporate cheese-ripening enzymes to milk in order to accelerate cheese proteolysis [50,51]. The rate of release could be affected by some factors such as cheese fat content, being stimulated by increasing the fat content (0% to 20%) [52].

It is worth noting that nanovesicles are considered to have a promising future in the food industry. Indeed, several commercialized liposome products have already been approved for different applications including food supplements and food preservatives as well as products for pathogen and pesticide detection [53].

Overall, our results show that nanovesicles are also suitable candidates for the production of phiIPLA-RODI-based formulations that will help to maintain phage stability during food processing conditions or in the gastrointestinal tract. More specifically, our results thus far seem to indicate that niosomes are the most interesting nanovesicles for the encapsulation of phiIPLA-RODI. Indeed, in addition to offering the greatest protection to the phage particles, they are also less costly than liposomes [40]. Future work will validate the effectiveness of encapsulated phages in a food matrix as long as the niosomes synthesis procedures are ready for scaling up, in order to make these formulations a real alternative to the food industry.

## Figures and Tables

**Table 1:** Composition, characteristics (homogenization and duration, size, zeta potential) and viability (measured as log reduction in phage titer after treatment comparing with the initial titer) of vesicles containing phage phiIPLA-RODI.

Vesicles	Composition		Homogenization (rpm)/Duration (min)	Z-Average (µm)	ζ-Potential (mV)	Viability Loss (Log Units)
	Components	Concentration *				
Niosome	Pronanosome Nio-N™	30 mg/mL,	8000/5	0.83 ± 0.11	−34.3 ± 1.0	0.5 ± 0.1
		50 mg/mL, or		0.85 ± 0.12	−33.4 ± 0.2	1.0 ± 0.2
		70 mg/mL		0.80 ± 0.07	−35.6 ± 1.3	1.1 ± 0.2
Liposome	Pronanosome Lipo-N™	30 mg/mL,	5000/5	1.51 ± 0.17	−14.1 ± 1.0	1.3 ± 0.1
		50 mg/mL, or		1.60 ± 0.17	−14.1 ± 0.5	1.2 ± 0.2
		70 mg/mL		1.89 ± 0.03	−13.5 ± 0.1	1.2 ± 0.2
Transfersome	Phospholipon 90G and Span 60 (1:1)	30 mg/mL,	8000/5	0.51 ± 0.07	−30.3 ± 1.2	1.4 ± 0.3
		50 mg/mL, or		0.55 ± 0.03	−30.8 ± 2.0	1.2 ± 0.0
		70 mg/mL		0.58 ± 0.06	−28.6 ± 0.2	1.3 ± 0.1

Notes: Each value represents the mean ± standard deviation of three samples. * In the case of transfersomes, the concentration refers to the total of the two components.

**Table 2:** Encapsulation efficiency for bacteriophage phiIPLA-RODI in niosomes, liposomes and transfersomes prepared using different concentrations of components.

Concentration of Components (mg/mL)	Encapsulation Efficiency (% PFU/mL)		
	Niosomes	Liposomes	Transfersomes
30	99.8.0 ± 0.03	98.6 ± 0.47	76.9 ± 21.49
50	94.5 ± 3.29	95.2 ± 4.30	95.6 ± 4.96
70	62.3 ± 14.35 *^,#^	85.5 ± 9.04	96.6 ± 2.89

Notes: Each value represents the mean ± standard deviation of three samples. The asterisk (*) indicates a significantly different efficiency of encapsulation as a function of the concentration and the pound (^#^) as a function of the type of nanovesicle (*p* < 0.05; ANOVA).

**Table 3:** Stability (log_10_ PFU/mL) of bacteriophage phiIPLA-RODI to different treatments.

Nanovesicles	Component (mg/mL)	Phage phiIPLA-RODI	Initial Titer log_10_ (PFU/mL)	pH 4.5 60 min	Tª 60 °C 90 min	NaCl 4.5 M 60 min
Niosomes	30	F	3.73 ± 0.20	−		4.74 ± 0.08 *
		E	5.78 ± 0.03	3.78 ± 0.1 *	3.54 ± 0.50 *	4.52 ± 0.13 *
		T	5.78 ± 0.03	3.78 ± 0.1 *	3.54 ± 0.50 *	4.95 ± 0.02 *
	50	F	4.09 ± 0.12	−	N/A	5.32 ± 0.18 *
		E	5.76 ± 0.07	4.00 ± 0.19 *	N/A	4.50 ± 0.10 *
		T	5.77 ± 0.06	4.00 ± 0.19 *	N/A	5.38 ± 0.15 *
	70	F	5.11 ± 0.07	−	N/A	5.79 ± 0.17 *
		E	5.63 ± 0.06	3.85 ± 0.16 *	N/A	4.44 ± 0.11 *
		T	5.74 ± 0.04	3.85 ± 0.16 *	N/A	5.81 ± 0.16
Liposomes	30	F	3.53 ± 0.15	−	−	5.03 ± 0.32 *
		E	5.80 ± 0.85	−	−	4.85 ± 0.22
		T	5.80 ± 0.84	−	−	5.25 ± 0.25
	50	F	4.16 ± 0.09	−	N/A	5.05 ± 0.12 *
		E	5.14 ± 0.51	3.46 ± 0.28 *	−	4.03 ± 0.37 *
		T	5.18 ± 0.22	3.46 ± 0.28 *	N/A	5.19 ± 0.14
	70	F	3.87 ± 0.26	−	N/A	4.71 ± 0.68
		E	5.03 ± 0.22	2.30 ± 0.24 *	N/A	4.48 ± 0.54
		T	5.06 ± 0.18	2.30 ± 0.24 *	N/A	4.91 ± 0.62
Transfersomes	30	F	−	−	−	4.93 ± 0.53 *
		E	4.88 ± 0.21	−	−	4.43 ± 0.34
		T	4.88 ± 0.21	−	−	5.01 ± 0.48
	50	F	−	−	N/A	5.01 ± 0.43 *
		E	4.63 ± 0.15	−	N/A	4.18 ± 0.22 *
		T	4.63 ± 0.15	−	N/A	4.75 ± 0.52
	70	F	2.95 ± 0.02	−	N/A	5.05 ± 0.19 *
		E	4.93 ± 0.05	−	N/A	4.60 ± 0.05 *
		T	4.94 ± 0.10	−	N/A	5.18 ± 0.17
Control phage in SM buffer	N/A	N/A	8.52 ± 0.10	−	4.75 ± 0.22 *	8.08 ± 0.48

Note: Each value represents the mean ± standard deviation of three samples. (F): free or non-encapsulated phage; (E): encapsulated phage; (T): Total phage = F + E. Initial titer: titer of phage before treatment process. (−) Below bacteriophage threshold (10^2^ PFU/mL). The asterisk (*) indicates a statistically significant difference with respect to the initial titer. N/A: not applicable.
